# Strain-engineered diffusive atomic switching in two-dimensional crystals

**DOI:** 10.1038/ncomms11983

**Published:** 2016-06-22

**Authors:** Janne Kalikka, Xilin Zhou, Eric Dilcher, Simon Wall, Ju Li, Robert E. Simpson

**Affiliations:** 1 Singapore University of Technology and Design (SUTD), 8 Somapah Road, Singapore 487372, Singapore; 2 Massachusetts Institute of Technology (MIT), Cambridge, Massachusetts 02139, USA; 3 Institut de Ciencies Fotoniques (ICFO), Barcelona Institute of Science and Technology, Castelldefels, Barcelona 08860, Spain

## Abstract

Strain engineering is an emerging route for tuning the bandgap, carrier mobility, chemical reactivity and diffusivity of materials. Here we show how strain can be used to control atomic diffusion in van der Waals heterostructures of two-dimensional (2D) crystals. We use strain to increase the diffusivity of Ge and Te atoms that are confined to 5 Å thick 2D planes within an Sb_2_Te_3_–GeTe van der Waals superlattice. The number of quintuple Sb_2_Te_3_ 2D crystal layers dictates the strain in the GeTe layers and consequently its diffusive atomic disordering. By identifying four critical rules for the superlattice configuration we lay the foundation for a generalizable approach to the design of switchable van der Waals heterostructures. As Sb_2_Te_3_–GeTe is a topological insulator, we envision these rules enabling methods to control spin and topological properties of materials in reversible and energy efficient ways.

## Abstract (web summary)

Strain engineering allows design of materials with tailored properties. Here, the authors show that strain can be used to control atomic diffusion in Sb_2_Te_3_-GeTe superlattices, and they propose general design rules to enable atomic switching functionalities in van der Waals heterostructures.

## Graphical abstract

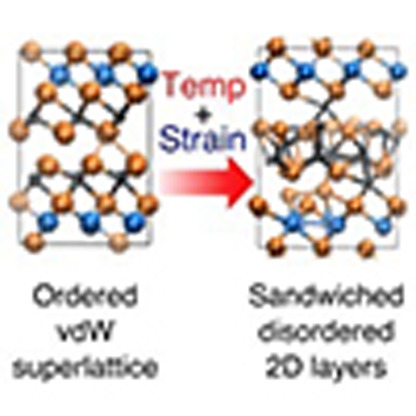

van der Waals (vdW) heterostructures composed from layers of two-dimensional (2D) atomic crystals^1^ provide unprecedented freedom to systematically design the properties of materials. Their properties are dependent on the sequence of the layers^2^, interlayer separation^3^ and the stress in the layers^4^. The crystal structure of Sb_2_Te_3_, which comprises of covalently bonded Sb–Te quintuple-layer blocks and vdW inter-block bonding, provides the framework to build Sb_2_Te_3_–GeTe vdW heterostructures. A systematic design methodology would accelerate the optimization of Sb_2_Te_3_–GeTe heterostructures, phase change materials and switchable topological insulators^5^. In this work we show that biaxial strain selectively destabilizes GeTe layers within the Sb_2_Te_3_–GeTe heterostructure and allows diffusive atomic disordering within the GeTe layers. In practice we demonstrate that this Ge–Te diffusive switching is controlled by the thickness of the Sb_2_Te_3_ layers and confined to an interface just 5 Å thick.

Strain engineering is possibly the foremost generalizable methodology used to design materials with bespoke properties. The most familiar example is in metal oxide semiconductor field-effect transistors (MOSFETs)^6^ where the Si channel is strained by a lattice mismatch between it and the surrounding material^7^. The stress in the Si crystal alters the mobility of charge carriers and increases the switching speed of the MOSFET. The discovery of super strong 2D materials has unleashed strain engineering from the sub-percent levels of strain that can be sustained in bulk crystals to the extraordinarily large strains of 11 and 20%, which can be sustained in graphene^8^ and MoS_2_ (ref. 9), respectively. This has provided strain engineers with a new degree of freedom to create strain-tuneable electronic and optoelectronic materials^10^.

Strain engineering provides a conceptually simple means to control the properties of materials at an atomic level. Furthermore, it is well suited to computer-aided atomistic design of materials using tools such as density functional molecular dynamics (DF/MD). In contrast, modelling doped materials require a large simulation cell and hundreds of atoms to reach the low dopant concentrations that are typically used in experiments. Consequently, simulating doped materials is extremely computationally expensive. Models of strained crystals do not suffer from this problem. It is, therefore, surprising that strain engineering has not been applied to the development of Sb_2_Te_3_–GeTe phase change materials. This may be due to the misbelief that reversible switching in these materials is only possible by a melt–quench process, which would cause strain relaxation. Recently, a number of demonstrations have shown that switching in Sb_2_Te_3_–GeTe materials is possible without melting the crystal structure. For instance, the optical properties of the Ge_2_Sb_2_Te_5_ alloy can be switched without transiting through the molten phase^11^, and without loss of long-range order^12^, suggesting that only a subset of atoms need to move to change the material properties. Moreover, non-melt crystal–crystal transitions are possible in Sb_2_Te_3_–GeTe vdW heterostructures^13^ and now, in this work, we show theoretically and experimentally that biaxial strain can be used to reduce the switching energy of Sb_2_Te_3_–GeTe vdW heterostructures.

Crystalline Sb_2_Te_3_–GeTe superlattice structures have been designed to promote the movement of interfacial Ge atoms through a plane of Te atoms^13^. In effect the interfacial Ge and Te layers exchange position—an atomic switch. Ideally this atomic transition reduces entropic losses leading to a high efficiency, low-energy switching process^14^. After switching Sb_2_Te_3_–GeTe superlattices, the electrical resistivity increases by several orders of magnitude yet the superlattice remains crystalline. This led to the suggestion that the material undergoes a crystal–crystal transition^13^. It is important to realize that this switching concept is fundamentally different to that in GeTe/Sb_2_Te_3_ superlattice-like structures (SLL)^15^. Both the GeTe and Sb_2_Te_3_ layers within the as-deposited SLL are relatively thick amorphous films that crystallize at different temperatures^16^ through three-dimensional diffusion of atoms into a system of polycrystalline^17^ layers without preferred orientation. The SLL's switching energy reduction stems from its low thermal conductivity^15^ and associated heating efficiency, not from controlled interfacial atomic diffusion.

The aim of this work is to establish a set of general rules that can be used to design and optimize phase transitions in vdW superlattices. Others have tried to further optimize switching in Sb_2_Te_3_-based superlattices by changing the composition of the other superlattice layers^18^,^19^,^20^, but the effects of doping and compositional tuning are difficult to predict and establishing general conclusions is not possible. In contrast, we model and experimentally demonstrate strain engineering of the Sb_2_Te_3_–GeTe superlattice such that the GeTe layers disorder at a temperature substantially lower than the melting temperature of the whole superlattice. Strain engineering is a more general approach to materials design than compositional tuning. Moreover, the approach can in principle be used to add switching function to vdW superlattices composed of other 2D crystals.

## Results

### Activation energy for switching

**Figure 1:**
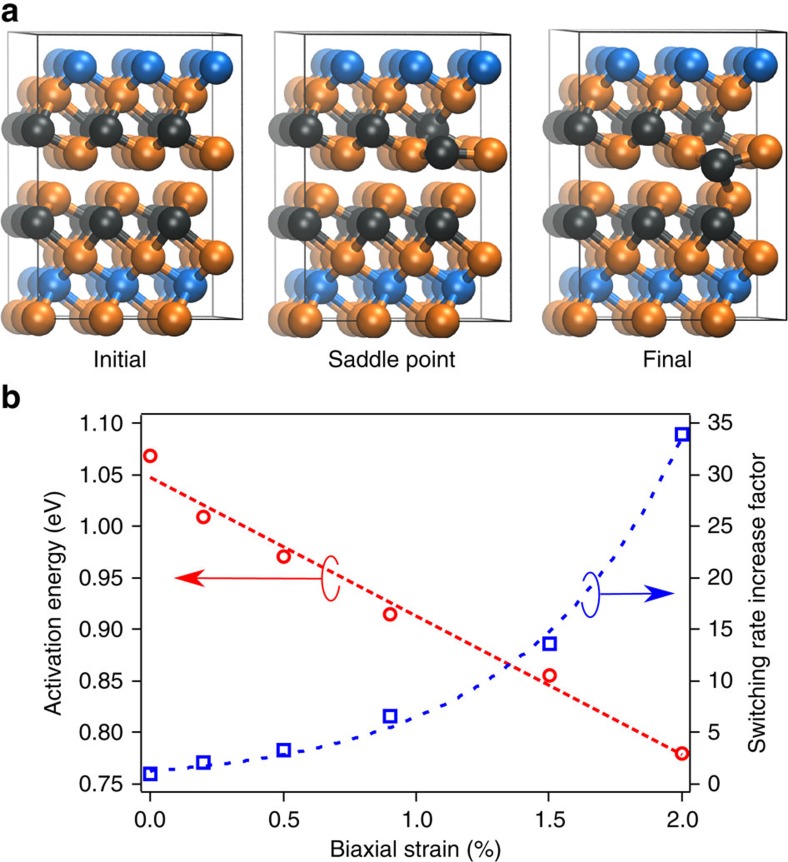
The calculated activation energy for a single Ge atom to move from the GeTe layer into the van der Waals gap. (**a**) The 81 atom model at different stages of the atomic transition. The initial, intermediate saddle point and the final structures for the Ge atomic transition are shown, where Ge, Sb and Te atoms are coloured black, blue and orange, respectively. The extent to which the atoms are shaded indicates depth in the simulation cell. The black box is the periodic boundary of the simulation cell. (**b**) The activation energy (red) for the transition is plot as function of biaxial strain. The increase in switching probability at 950 K relative to the unstrained superlattice is also plot as a function of biaxial strain (blue).

Recently Yu and Robertson^21^ showed that the activation energy for switching of Ge atoms in the Sb_2_Te_3_–GeTe superlattice is dominated by the vertical movement of Ge atoms through a plane of Te atoms. By calculating the activation energy for a single Ge atom to switch through the planar Te layer using the nudged elastic band transition state search method, which is illustrated in Fig. 1a, we find that the activation energy decreases linearly as the biaxial tensile strain is increased. Consequently the atomic switching probability at 950 K for the structure biaxially strained by 1.5% increases by an order of magnitude relative to the unstrained structure, see Fig. 1b. The calculated reduction in activation energy for atomic switching assumes that the Sb_2_Te_3_ and GeTe layers are stable but in reality a high temperature, close to the structure's melting point, is required for a significant proportion of the interfacial atoms to undergo atomic switching.

### Applying strain to layers in a van der Waals superlattice

Sb_2_Te_3_ has a larger in-plane lattice constant than GeTe. To assess the tensile strain applied to the GeTe layers of Sb_2_Te_3_–GeTe superlattices, we grew different superlattice structures with the Sb_2_Te_3_ layer thickness varying from 1 to 4 nm, whilst the GeTe layers' thickness was fixed at 1 nm. The superlattice lattice parameters were measured by X-ray diffraction in a symmetric geometry. Only the (00L) peaks were present in the diffraction patterns thus indicating highly textured, layered superlattice films, see Supplementary Fig. 1 and Supplementary Note 1. The measured lattice parameters for GeTe in a hexagonal setting^13^,^22^ were used to calculate the biaxial tensile strain in the Sb_2_Te_3_ and GeTe layers relative to their bulk values, see Supplementary Fig. 2. Table 1 shows that as the Sb_2_Te_3_ layer thickness is increased, the biaxial strain in the GeTe layer increases from 0.72 to 2.16%. By comparing these measured strain values with the activation energy, which is shown in Fig. 1, it is clear that the activation energy for switching can be greatly reduced by increasing the thickness of the Sb_2_Te_3_ layers. Therefore a lower switching energy is to be expected for Sb_2_Te_3_–GeTe structures with thicker Sb_2_Te_3_ layers.

### Premelt disordering in strained van der Waals superlattices

**Figure 2:**
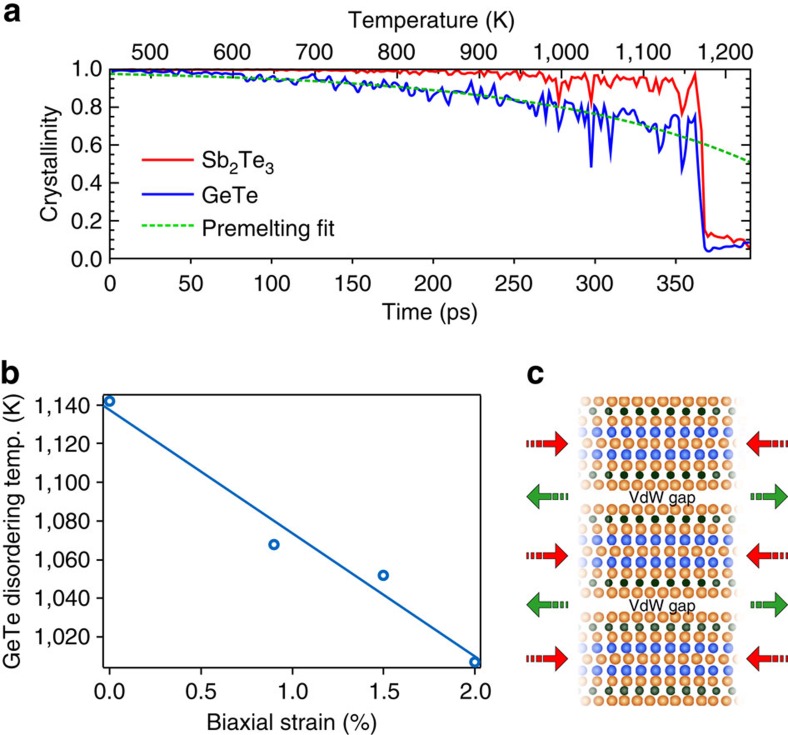
Strain-controlled interfacial premelting. (**a**) The crystallinity evolution for the 2% biaxially strained Sb_2_Te_3_–GeTe vdW superlattice. The crystallinity of the Sb_2_Te_3_ layers (red) remains close to 1.0 until the superlattice structure melts. In contrast, the GeTe layers (blue) premelts at ∼160 K below the melting point of the Sb_2_Te_3_ layer. The dashed green line is a fit to the GeTe crystallinity and was used to determine the temperature that the crystallinity dropped to 0.8, which we defined as the disordering temperature. Similar plots for 0% strain, 0.9 and 1.5% biaxial strain are given in Supplementary Fig. 3. (**b**) The disordering temperature of the GeTe layers is plot as a function of biaxial strain in the GeTe layers. Increasing the biaxial strain decreases the GeTe disordering temperature. (**c**) Relaxed Sb_2_Te_3_–GeTe vdW heterostructure. The direction of strain in the 2D crystal lattices alternates between tensile (GeTe) and compressive (Sb_2_Te_3_) relative to their respective bulk crystal lattices. The red and green arrows indicate the layers subjected to compressive and tensile strain respectively. The atomic colours are: Ge atoms—black, Sb atoms—blue, and Te atoms—orange.

It is clear from Table 1 that when a 2D GeTe crystal is incorporated in an Sb_2_Te_3_–GeTe superlattice heterostructure, the GeTe crystal is strained and therefore distorted with respect to the bulk GeTe crystal. We therefore investigated the effect of biaxial strain on the stability of the GeTe layers within the Sb_2_Te_3_–GeTe heterostructure by DF/MD simulations. The crystallinity of the individual GeTe and Sb_2_Te_3_ layers was calculated as a function of time. The crystallinity evolution of the individual Sb_2_Te_3_ and GeTe layers for the 2% biaxially strained superlattice is shown in Fig. 2a whilst the crystallinity plots for other strains are given in Supplementary Fig. 3 and discussed in Supplementary Note 2. Both the Sb_2_Te_3_ and the GeTe layers show an abrupt decrease in crystallinity at a similar melting temperature. However, the GeTe crystallinity gradually decreases as the temperature increases above 600 K. In effect, premelt disordering starts to occur in a 5 Å thick GeTe 2D plane at a temperature 300 K below the melting temperature of the whole structure. Although melting is stochastic by nature, there is a clear trend that increasing biaxial strain decreases this GeTe premelt disordering temperature, as shown in Fig. 2b. Thus we conclude that disordering in the GeTe layers of the superlattice is highly sensitive to biaxial strain, which can be controlled by changing the Sb_2_Te_3_ layers' thickness.

### Diffusive atomic switching in vdW superlattices

**Figure 3:**
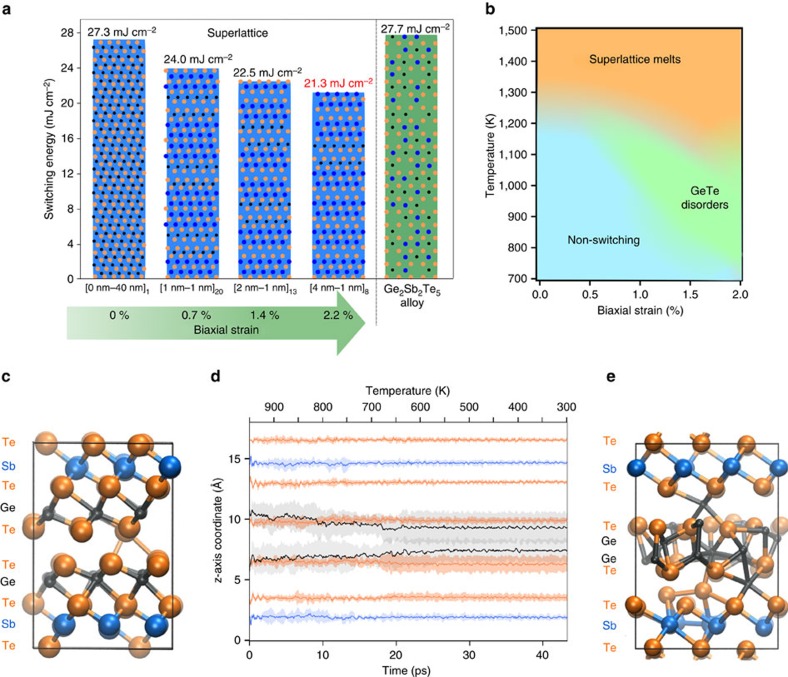
GeTe disordering. (**a**) The measured minimum laser fluence required to decrease the optical reflectivity of the Sb_2_Te_3_–GeTe vdW superlattices is shown for different thicknesses of the Sb_2_Te_3_ layers (blue coloured bars). The biaxial strain in the GeTe layers increases with the Sb_2_Te_3_ layer thickness, see Table 1, resulting in a lower threshold fluence to induce disordering in the GeTe layers. The switching fluence required for the Ge_2_Sb_2_Te_5_ alloy is also shown (green coloured bar). The dots on the bars illustrate the atomic layer sequence where the Ge, Sb, and Te atoms are coloured black, blue, and orange respectively. (**b**) A strain–temperature map to show the regions where the GeTe and SbTe layers remain stable (blue area), and where diffusive Ge atomic disordering (green area) and superlattice melting (orange area) are likely. GeTe disordering is possible when the superlattice structure is strained. (**c**) The initial (Sb_2_Te_3_, 1 nm—GeTe, 1 nm) superlattice structure in the resonantly bonded crystalline state. (**d**) The mean *z*-coordinate evolution of the atoms in each layer during a simulated quench from 950 K for the 1.5% strained superlattice structure (two similar quenches from 950 and 1,400 K for the 0% strained superlattice are included in Supplementary Figs 7 and 8, and supporting information is given in Supplementary Note 4). The solid lines show the mean position of the nine atoms in each layer where Ge, Sb and Te atoms are coloured black, blue and orange, respectively. The semitransparent shading indicates the standard deviation, ±*σ*, in the *z*-coordinate and was calculated according to 
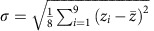
, where 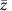 is the mean *z*-coordinate of the nine atoms in each layer and *i* is the atom index in the layer. (**e**) The structure after quenching to 300 K. The Sb_2_Te_3_ layers remain in tack after the quench, whilst diffusive atomic disordering is confined to a 5 Å thick two-dimensional layer of GeTe layer. Colours: Ge–black, Sb–blue and Te–orange.

We used femtosecond laser pulses to measure the energy required to switch the superlattices into a low reflectivity state. Single 30 fs laser pulses at a wavelength of 800 nm were used to switch 40 nm thick Sb_2_Te_3_–GeTe crystalline superlattice samples into a low reflectivity state. The minimum laser fluence required to write a low reflectivity spot into the film is shown as a function of strain in Fig. 3a. An example of the laser fluence measurement is shown in Supplementary Fig. 4. Increasing the biaxial strain in the GeTe layers by increasing the Sb_2_Te_3_ layer thickness reduces the fluence necessary for switching the optical properties of the structure. For reference, the measured switching energy for cubic Ge_2_Sb_2_Te_5_ is also shown. It is worth noting that the superlattice composed of 1 nm thick Sb_2_Te_3_ layers and 1 nm thick GeTe layers has the average composition Ge_2_Sb_2_Te_5_. However, its threshold switching energy is lower than the Ge_2_Sb_2_Te_5_ alloy, which shows that the superlattice structure can be used to design and optimize switching in Sb_2_Te_3_–GeTe-based materials. Moreover, the GeTe layers in superlattices composed of 1 nm thick Sb_2_Te_3_ layers and 1 nm thick GeTe layers are subjected to almost 1% biaxial strain relative to the unstrained 40 nm thick GeTe film, see Table 1. We see in Fig. 3a that this also substantially reduces the threshold switching energy relative to the crystalline GeTe film.

To confirm the reversibility of the switching process the optical reflectivity of the laser switched marks was measured before and after annealing the samples at 220 °C. The optical reflectivity for all the samples typically increased by 15% after annealing such that the switched areas were indistinguishable from the background crystalline film. This indicates the reversibility of the GeTe disordering process (Supplementary Figs 5 and 6).

To establish the influence of strain and temperature on the relative ability for the GeTe layer to diffusively disorder, we used DF/MD to map the strain–temperature parameter space and find the conditions required for Ge atoms to diffuse into the vdW gap. Recent time resolved structural measurements of femtosecond laser amorphisation of crystalline Ge_2_Sb_2_Te_5_ showed that the melting process is typically complete in 5 ps (ref. 12). Therefore for different levels of biaxial strain we estimated the required temperature to switch at least one Ge atom into the vdW gap in 5 ps. The map shown in Fig. 3b, which is detailed in Supplementary Table 1 and Supplementary Note 3, shows that the unstrained structure is stable up to 1,300 K and Ge atomic switching is unlikely during the 5 ps simulation. When the temperature is raised to 1,400 K, the Sb_2_Te_3_ and GeTe layers melt and inter-diffuse. However, we find that biaxial tensile strain greatly enhances Ge–Te atomic switching by premelting the 5 Å thick GeTe layer. When the Sb_2_Te_3_–GeTe heterostructure is biaxially strained by 0.9%, Ge–Te diffusive atomic switching occurs at temperatures below the superlattice melting temperature. Further increases in tensile biaxial strain widens the temperature window for diffusive atomic switching.

The stability of the switched phase was studied with long quench runs where the structure was heated to temperatures ranging from 700 to 1,400 K, and cooled to 300 K at a rate of −15 K ps^−1^ to mimic the effect of a short laser heating pulse and subsequent thermal dissipation. Diffusive Ge–Te atomic switching can be observed by plotting the *z*-coordinate of the atoms in the simulation cell as a function of time and temperature, see Fig. 3d. Ge–Te atomic switching is visualized as an exchange of the Ge and Te relative layer positions and a crossing of their *z*-coordinates in the evolution plot. If the structure melts, the atomic layers inter-diffuse and the resultant plot does not show the regular *z*-coordinate spacing that is observed for a layered solid. Examples of *z*-coordinate evolution plots for 0% strain that are quenched from different temperatures are given in Supplementary Figs 7 and 8, and described in Supplementary Note 4.

Figure 3c–e shows the structural evolution of the Sb_2_Te_3_–GeTe heterostructure that was subjected to 1.5% biaxial strain and an initial temperature of 950 K. After ∼10 ps most of the Ge atoms have moved into the Te–Te vdW gap producing disordered 2D GeTe layers encapsulated by crystalline Sb_2_Te_3_ quintuple blocks. The bottom layer Ge atoms completely switch into the gap within approximately 1 ps, however this is likely to be an artefact of the molecular dynamics initial conditions, and therefore we focus on the movement at 10 ps where the system has had some time to equilibrate. By quenching the structure at −15 K ps^−1^, the switched Ge atoms are frozen between the Te–Te layers and this is visible as a dampening of the atomic movement about their equilibrium *z*-position. The structure retains a layered nature after the diffusive switching.

## Discussion

We have shown that the activation energy for Ge atoms to diffuse into the vdW gap (Fig. 1b), the temperature for premelt disordering of the GeTe layers (Fig. 2b), and the switching energy required to observe a reduction in optical reflectivity (Fig. 3a) are all lowered by biaxially straining the superlattice GeTe layers. Applying biaxial tensile strain to the Sb_2_Te_3_–GeTe interface increases the planar separation of Te atoms and reduces the energy barrier for the Ge atoms to move through the Te plane, and this explains the measured lower switching energy.

In practice it is common to strain crystalline films by heteroepitaxial growth from a substrate with a small lattice mismatch, or by capping with a stressed film. However, these techniques do not apply to vdW bonded structures because the in-plane lattice constants of vdW solids usually relax to the bulk values after three vdW interfaces^23^. However, we use an Sb_2_Te_3_–GeTe superlattice with Sb_2_Te_3_ layers less than four quintuple blocks thick. This means the GeTe layers reset the strain in the Sb_2_Te_3_ layers before it can fully relax. Since the *a* lattice parameter of the bulk Sb_2_Te_3_ crystal is 2.4% larger than that of bulk GeTe, it is possible to biaxially strain the GeTe layer by increasing the thickness of the Sb_2_Te_3_ layers within the superlattice. We find that the superlattice takes a weighted average in-plane lattice parameter of the GeTe and Sb_2_Te_3_ bulk crystal lattice parameters.

Ideally, as the strain applied to the sample increases, the Sb_2_Te_3_ scaffold layers should remain stable whilst the GeTe layers should become increasingly unstable, which is necessary for diffusive atomic switching. This requires a bond hierarchy to exist within the heterostructure. Crystalline GeTe and related phase change materials, such as Ge_2_Sb_2_Te_5_, have an extraordinarily large optical refractive index compared with that of their amorphous structure. This is due to the existence of highly polarizable *p*-orbital electrons in crystalline GeTe and less polarizable bonds in the amorphous state^24^. The GeTe crystal is characterized by a rhombohedral unit cell with the Ge atom lying off-centre along the [111] direction to give three long, and three short Ge–Te bonds, and therefore a bond energy hierarchy exists for this crystal. DFT calculations have shown that electrons are delocalized across multiple unit cells along the longer Ge–Te bonds^25^, which explains its high polarizability and concomitantly large refractive index. This type of delocalized bonding is commonly referred as ‘resonance bonding' and was introduced in the context of IV–VI compounds, including GeTe, by Lucovsky and White^26^. In contrast, the shorter GeTe bonds are localized at the mid-point between the Ge and Te atoms. The stability of GeTe is dependent on the rather fragile alignment of resonant bonds across multiple unit cells; hence the GeTe crystal is particularly sensitive to structural distortions^25^.

The GeTe premelt disordering, which is shown in Fig. 2a,b, at a temperature below the melting temperature of Sb_2_Te_3_ may seem surprising when considering the higher bulk melting temperature of GeTe (997 K)^27^, compared with Sb_2_Te_3_ (891 K)^28^. However, we highlight again the sensitivity of the GeTe crystal to distortions^25^. For small distortions, such as those encountered by straining the GeTe lattice, resonance bonding is weakened but not broken. We see from Table 1 that as the thickness of the Sb_2_Te_3_ layer is increased, the GeTe lattice is put under biaxial tensile strain and consequently the Ge–Te resonant bonds are weakened and the disordering temperature of the GeTe layers is lowered. In contrast, the lattice parameter of the Sb_2_Te_3_ layers tend to the bulk value and therefore the layers remain crystalline, see Fig. 2a and Supplementary Fig. 3. In effect, the unstrained superlattice structure consists of biaxially compressed Sb_2_Te_3_ and biaxially tensile strained GeTe, which effectively increases the stability of the Sb_2_Te_3_ layer whilst decreasing the stability of the GeTe layer. This situation is illustrated in Fig. 2c. Consequently, increasing the Sb_2_Te_3_ thickness increases the strain in the GeTe layer, destabilizing it and promoting premelt disordering. The reduction in the laser energy used to disorder the GeTe layers, which is shown in Fig. 3a, is due to strain induced destablization of resonant bonds. This results in preferential disordering of GeTe layers at temperatures below the superlattice melting temperature. The disordered GeTe layers produce a superlattice structure with a lower optical reflectivity. This is to be expected since disorder forbids resonant bonding, which is responsible for the extraordinarily large refractive index of the GeTe crystal. The high reflectivity, switched, state was readily recovered for all structures after annealing the samples at 220 °C. Supplementary Figs 6 and 9 show that both the measured and modelled reflectivity modulation is ∼10% in the visible spectrum (further details are given in Supplementary Notes 5 and 6). It is worth noting that phase change memory cells composed of similar superlattice structures exhibit excellent cycleability^13^ and we expect that these strained superlattices should also be highly cycleable between the high and low reflectivity states.

Figure 3c–e shows that the Sb_2_Te_3_ layers can remain crystalline during the GeTe disordering. During the 43 ps simulation Sb_2_Te_3_ atoms retained their crystallographic positions. Disordering is confined to the GeTe layers that are less than 5 Å thick—a 2D phase transition occurs. The final ‘switched' structure is shown in Fig. 3e. While the activation energy for a single Ge atom penetrating the Te plane is lowered by applying tensile strain, see Fig. 1b, we now also see a tendency for the GeTe layer to disorder after the transition. Note that this switching behaviour is in stark contrast to the GeTe layer in the unstrained structure at 950 K, which does not disorder and all atoms remain in their initial unswitched crystallographic positions. At higher temperatures the Sb_2_Te_3_ layers in the unstrained superlattice are unstable and both the GeTe and Sb_2_Te_3_ layers inter-diffuse, see Supplementary Figs 7 and 8.

From these results we conclude that diffusive Ge–Te atomic switching in Sb_2_Te_3_–GeTe vdW heterostructures can be thermally stimulated by premelting the GeTe layer at temperatures lower than the melting point of Sb_2_Te_3_. Increasing the thickness of the Sb_2_Te_3_ layer increases the biaxial strain in the GeTe layer and causes a related increase in the atomic switching probability and a decrease in the switching energy. The Sb_2_Te_3_ layers experience little distortion during the switch and their rigidity is crucial for the stability of the whole superlattice structure. As such, Sb_2_Te_3_ presents a versatile scaffold to strain engineer the properties of GeTe. We conclude that in-plane biaxial strain significantly enhances diffusive atomic disordering within the GeTe layers of Sb_2_Te_3_–GeTe superlattices.

Herein, diffusive atomic switches based on strain-engineered Sb_2_Te_3_–GeTe heterostructures have been developed. However, we believe that the applicability of strain engineering can be generalized and extended to other vdW bonded heterostructures of 2D chalcogenide crystals and therefore we highlight the following rules that can be used to design similar diffusive atomic switching heterostructures: First, the switching material (GeTe) in the heterostructure must exhibit resonant bonding such that its structure is sensitive to distortions and strain can be used to modify the disordering energy. Second, the scaffold material (Sb_2_Te_3_) in the heterostructure must be stable over the temperature range that the switching material exhibits premelting. Third, the scaffold material must have a larger bulk lattice parameter, *a*, than the switching material. Fourth, it must be possible to grow the vdW heterostructure superlattices from 2D crystals of the switching and scaffold materials by vdW heteroepitaxy such that the layers strain one another.

The first point prevents these design rules being applied to phase change materials with tetrahedral crystal structures, such as Cu_2_GeTe_3_ (ref. 29) and GaSb (ref. 30), where resonant bond alignment across multiple unit cells is not possible. The third point is necessary such that the switching material is under tensile strain relative to its bulk, and therefore exhibits a melting temperature that is controlled by the thickness of the scaffold layers.

The ability to tune the strain profile of vdW heterostructures opens a further degree of freedom to manipulate their function and properties. We have demonstrated that diffusive disordering and atomic switching can occur by selectively destabilizing 5 Å thick GeTe 2D crystal layers within a Sb_2_Te_3_–GeTe vdW heterostructure superlattice. The extent of the destablization is controlled by the thickness of the Sb_2_Te_3_ layers, which strain the GeTe layers. Consequently, thicker Sb_2_Te_3_ layers cause a significant decrease in the energy necessary for switching the properties of Sb_2_Te_3_–GeTe superlattices. More generally, four rules have been identified that can be used to aid the design of 2D crystal vdW heterostructures with switchable properties.

## Methods

### Computational methods

All DF/MD runs were performed using the Vienna Ab initio Simulation Package (VASP 5.3.3)^31^ with PAW-pseudopotentials^32^, PBE exchange-correlation functional^33^, 3 fs timestep, and an NVT ensemble with periodic boundary conditions. For the superlattice structure geometry optimizations at 0 K, which were used to calculate the superlattice lattice parameters given in Table 1, we used a hexagonal unit cell with a Γ centred mesh of 8 × 8 × 2 points. We used a plane-wave basis with 240 eV cutoff. The energy precision for the self consistent field calculation was set to 10^−6^ eV.

To model the effect of biaxial strain on diffusive atomic transitions, an 81 atom Sb_2_Te_3_–GeTe model was built. For the DF/MD runs energies were calculated at the Γ point of the Brillouin zone (**k**=0), which led to an accuracy of 50 meV per atom relative to a 5 × 5 × 5 k-point mesh. We used a plane-wave basis with 220 eV cutoff. The energy precision for the self consistent field calculation was set to 10^−5^ eV. The temperature of the model was controlled by velocity rescaling. The superlattice unit cell dimensions were estimated by running 10 ps of MD at 300 K with different cell dimensions. The 300 K relaxed cell dimensions were then found by selecting the structure with the lowest square-average stress tensor diagonal elements over the last 5 ps of the trajectory. This cell was then elongated by the desired strain amount in the *xy*-plane, and similar 10 ps simulations were performed to find the relaxed *z*-length while keeping the lateral strain.

Crystallinity was measured with bond orientational (BO) order parameter of Steinhardt *et al*.^34^, which was also used previously in crystallization simulations of Ge_2_Sb_2_Te_5_ (ref. 35). The BO order parameter was calculated by projecting the bond vectors onto a basis of spherical harmonics *Y*_*lm*_, with a suitable *l* value. The first nonzero *l* value for cubic lattice, *l*=4 was used. The order parameter **Q**_*l*_ is defined as


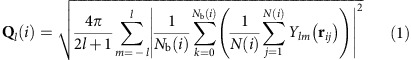


where **r**_*ij*_ is the vector between atoms *i* and *j*, *N*(*i*) is the number of neighbours for atom *i*, and *N*_b_ includes the atom *i* and its neighbours. The **Q**_4_(*i*) value for ideal, vacancy-free rock-salt lattice is 0.764. To compensate for thermal fluctuations we defined a crystalline atom as having a **Q**_4_(*i*) magnitude greater than 0.6. This method results in a good agreement with the atoms marked as crystalline by the order parameter and the atoms that look crystalline in visualizations.

The premelting in the Sb_2_Te_3_–GeTe superlattice was studied with a temperature ramp for each strain. The initial temperature was 450 K, and it was increased at 1.97 K ps^−1^ until the structure melted. The initial velocities were random, and scaled according to the temperature. The layer crystallinities were calculated as an average over all the atoms occupying the space initially occupied by each layer, and over 1 ps (1.97 K) windows.

The activation energy for a single Ge atom to switch into the vdW gap was calculated with the DFT code CASTEP^36^ using the 81 atom GeTe–Sb_2_Te_3_ model cell with ultra-soft pseudo-potentials. The plane-wave basis cutoff energy was set to 230 eV. The atomic positions and the cell shape was relaxed to the ground state using the BFGS^37^ minimizer with an energy tolerance of 5 × 10^−6^ eV per atom. The maximum force on the atoms was less than 0.01 eV Å^−1^. The energy was calculated at the gamma point. The atomic positions of the switched structure were also relaxed using the same criteria as listed above. The simulation cell volume was fixed with the non-switched volume of 2,501.63 Å^3^. The nudged elastic band method was then used to calculate the activation energy for a single Ge atom to switch into the vdW gap. A linear synchronous transit (LST) search was used to initially approximate the barrier height. The activation energy was then refined by cyclically repeating a quadratic synchronous transit (QST) maximization with a conjugate gradient minimization until the stationary point of the barrier was found^38^. The relative probability that the Ge atoms moved into the vdW gap was calculated using the Arrhenius equation 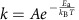, as a function of strain at 950 K.

Note that the DFT energy of our models was not corrected for the inter-block vdW interaction. Our strain-engineered switching model is based on the GeTe layer disordering process being dominated by the in-plane separation of Te–Te bonds rather than the out-of-plane vdW interactions. Table 1 shows that the error between the DFT modelled in-plane lattice parameter and the X-ray diffraction measured in-plane lattice parameter is <2%. Therefore the PBE exchange-correlation functional has use for modelling biaxially strained layered materials. Indeed, our simple 81 atom model proved to be an essential tool for designing superlattice structures with in-plane biaxial strain. Extending our models into a full simulation would involve accounting for vdW interactions, and fully simulating the (Sb_2_Te_3_, 2 nm—GeTe, 1 nm) and (Sb_2_Te_3_, 4 nm—GeTe, 1 nm) superlattice structures to find their in-plane lattice parameter, rather than modelling the larger lattice parameter by straining the (Sb_2_Te_3_, 1 nm—GeTe, 1 nm) structure, which we did here.

### Sb_2_Te_3_–GeTe superlattice growth

The (Sb_2_Te_3_, *x* nm—GeTe, 1 nm), where *x*=1, 2 and 4, superlattice films for laser switching measurements were grown on Si (100) substrates using layer-by-layer sputter deposition. Before the superlattice deposition the native oxide was removed from the Si substrate using a 30 W RF plasma for 60 min at a substrate holder temperature of 300 °C. A 10-nm thick Sb_2_Te_3_ buffer layer was then grown on the Si surface before the deposition of the superlattice film. The samples were grown in an Ar atmosphere of 0.5 Pa, whilst the base pressure of the vacuum chamber was better than 2.5 × 10^−5^ Pa. The substrate holder temperature was held at 300 °C during the superlattice growth. For all samples the total film thickness was kept constant at 40 nm and the composition was confirmed by energy dispersive X-ray (EDX) spectroscopy. The lattice parameters (*a*_SL_, *c*_SL_) of the superlattices were determined by Rietveld refinement. The in-plane biaxial strain applied to the GeTe and Sb_2_Te_3_ layers is dictated by the superlattice in-plane lattice parameters, and this was estimated by 
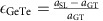
 and 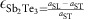, where *a*_GT_ and *a*_ST_ are the in-plane lattice constants for bulk GeTe and bulk Sb_2_Te_3_ in a hexagonal setting. To avoid encapsulation induced stresses^39^ all films were uncapped. To minimize oxidation the samples were either measured immediately after growth or stored under vacuum until the measurement was conducted.

### Switching of superlattice samples

Samples were mounted on a linear XY stage with the surface normal to the incident laser beam. The laser was focused to a Gaussian spot with a radius of 260 μm. A Pockels cell was used to extract a single femtosecond laser pulse on demand and the pulse energy was controlled by a half wave plate and polariser based attenuator. After each laser pulse, the sample was moved to a new position. The sample was then examined in a microscope to observe the phase transformation. Below a critical fluence *F*_A_ the laser did not induce a permanent change in the reflectivity. Above *F*_A_, the contrast between crystalline and amorphous phases grew until the contrast saturated and the lateral size of the switched area increased (sample images are provided in Supplementary Fig. 5). Further increasing the fluence eventually resulted in ablation and damage to the film. For further details see Supplementary Note 5.

### Data availability

The data that support the findings of this study are available from the corresponding author upon request.

## Additional information

**How to cite this article:** Kalikka, J. *et al*. Strain-engineered diffusive atomic switching in two-dimensional crystals. *Nat. Commun.* 7:11983 doi: 10.1038/ncomms11983 (2016).

## Supplementary Material

Supplementary Information Supplementary figures 1-9, Supplementary table 1, Supplementary notes 1-6 and Supplementary references.

## Figures and Tables

**Table 1:** Superlattice lattice parameters.

Layer thickness (Å)		Lattice parameter (Å)				In-plane biaxial strain (%)			
Sb_2_Te_3_	GeTe	X-ray diffraction		DFT		X-ray diffraction		DFT	
		a	c	a	c	Sb_2_Te_3_	GeTe	Sb_2_Te_3_	GeTe
Bulk	0	4.26	30.43	4.341	31.069	0.00	NA	0.00	NA
0	Bulk	4.16	10.66	4.237	10.887	NA	0.00	NA	0.00
10	10	4.19	19.38	4.268	17.832	−1.64	0.72	−1.68	0.73
20	10	4.22	30.12	4.269	28.522	−0.94	1.44	−1.66	0.76
40	10	4.25	50.11	4.311	49.201	−0.23	2.16	−0.69	1.75

DFT, density functional theory; NA, not applicable.

The Sb_2_Te_3_–GeTe superlattice lattice parameters for varying Sb_2_Te_3_ layer thicknesses are presented. These hexagonal lattice parameters were obtained by Rietveld fitting the X-ray diffraction patterns shown in Supplementary Fig. 1 and Supplementary Note 1. The DFT modelled lattice parameters are also shown. These lattice parameters were used to calculate the measured and modelled in-plane biaxial strain for the Sb_2_Te_3_ and GeTe layers relative to their bulk values.
