# Supplementary material for: Strain-engineered diffusive atomic switching in two-dimensional crystals
Source: Nat Commun. 2016 Jun 22;7:11983. doi: 10.1038/ncomms11983 (PMC4917972; doi:10.1038/ncomms11983)
Supplement: Supplementary Information — Supplementary figures 1-9, Supplementary table 1, Supplementary notes 1-6 and Supplementary references. [file ncomms11983-s1.pdf]

SUPPLEMENTARY FIGURES

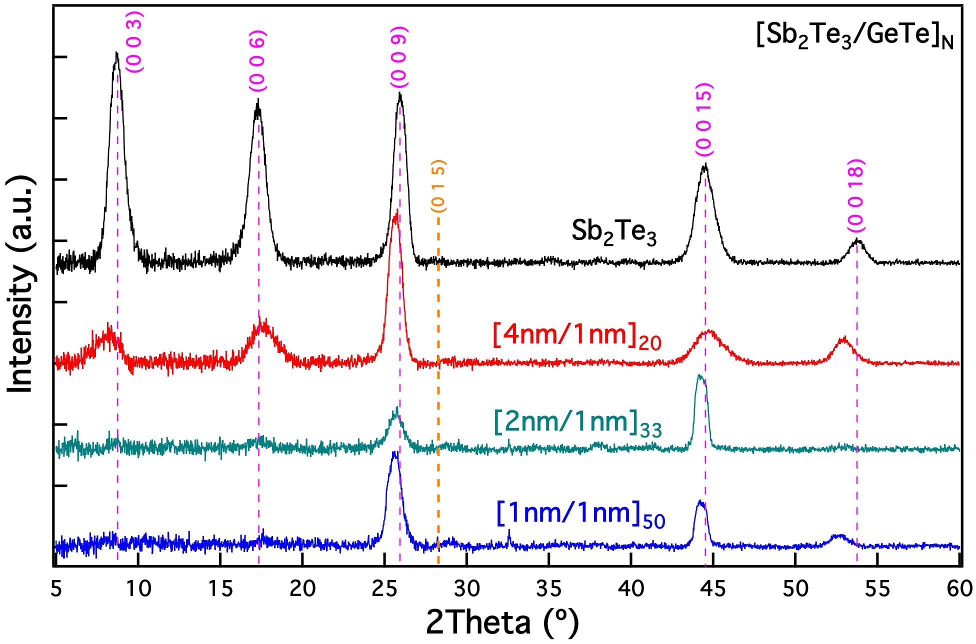

Supplementary Figure 1. X-ray diffraction patterns collected from the Sb_2Te_3–GeTe superlattice samples. Three different Sb_2Te_3–GeTe superlattice structures are shown and compared with Sb_2Te_3 (black). The notation $[4nm/1nm]_{20}$, $[2nm/1nm]_{33}$, and $[1nm/1nm]_{50}$ is used to describe the superlattice structure, where the first number is the thickness of the Sb_2Te_3 layers, the second number is the thickness of the GeTe layers, and the subscript describe the number of Sb_2Te_3-GeTe cycles are used to build the superlattice structure. The thickness of all superlattice films was 100 nm. For reference the diffraction angle of the Sb_2Te_3 (00L) planes are indicated in pink. We see that only (00L) peaks are present for these van der Waals heterostructure superlattice systems of Sb_2Te_3 and GeTe 2D crystals. See Supplementary Note 1 for further details.

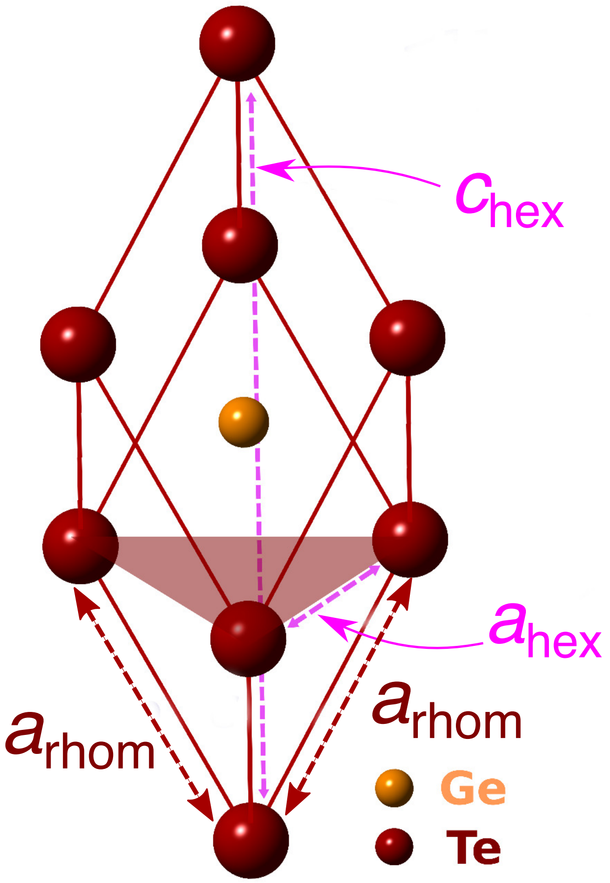

Supplementary Figure 2. Lattice parameters of the GeTe primitive cell as described in Ref.1. The 2D GeTe crystal layers are grown along the Sb_2Te_3 – GeTe superlattice $< 111 >$ direction of its rhombohedral primitive cell, which is aligned to the c-axis of a hexagonal Sb_2Te_3 crystal. In the rhombohedral setting the GeTe lattice parameters is $a_{\mathrm{rhom}} = 4.29$ Å. In the hexagonal setting the GeTe lattice parameters are $a_{\mathrm{hex}} = 4.16$ Å and $c_{\mathrm{hex}} = 10.66$ Å(ref.1). See Supplementary Note 1 for further details.

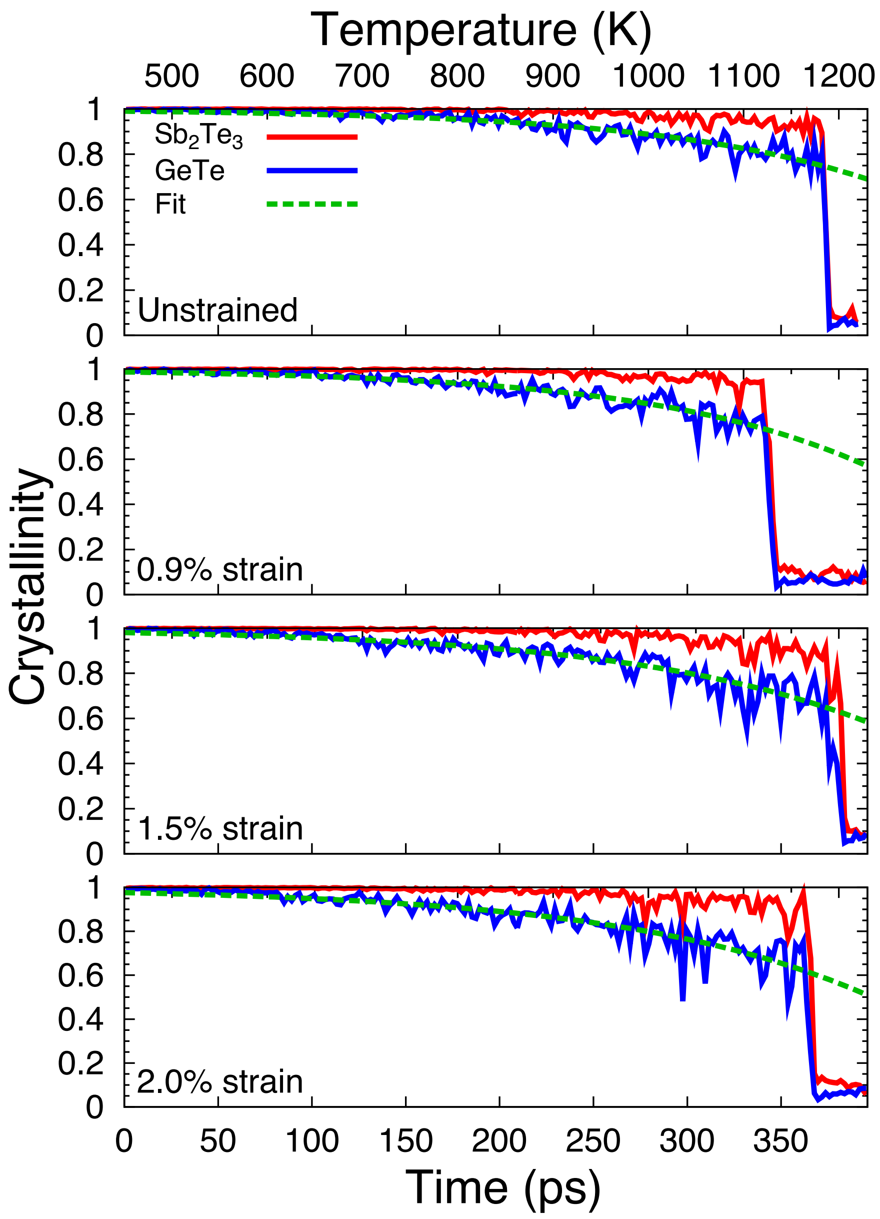

Supplementary Figure 3. The crystallinity of the GeTe and Sb_2Te_3 layers in the Sb_2Te_3–GeTe superlattice is plot as a function temperature for different amounts of in plane biaxial strain. In all cases the crystallinity of the Sb_2Te_3 layers remains close to 100% until the superlattice structure melts. In contrast, the GeTe disorders at temperatures substantially below the melting point of the superlattice. Increasing the amount of biaxial strain lowers the GeTe premelt disordering temperature. The dashed green line shows a fit to the GeTe crystallinity. The temperature at which the fit reaches a crystallinity of 0.8 was used to determine the disordering temperature of the GeTe layers. The disordering temperature as a function of biaxial strain is shown in Figure 2b of the main manuscript.

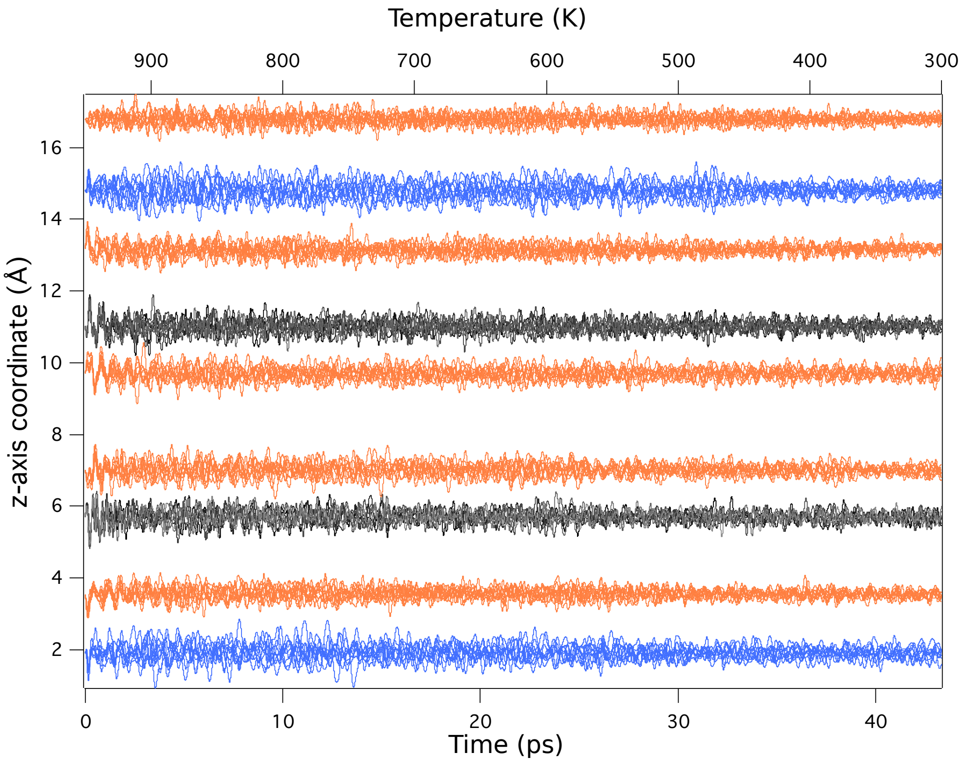

Supplementary Figure 4. The z-coordinate of atoms in unstrained superlattice structure when cooled from 950 K to 300 K. For this unstrained case, the layers do not melt or diffuse. The layer order is stable and does not change during the simulation. Colour code: Te –Orange, Sb –blue, Ge –black.

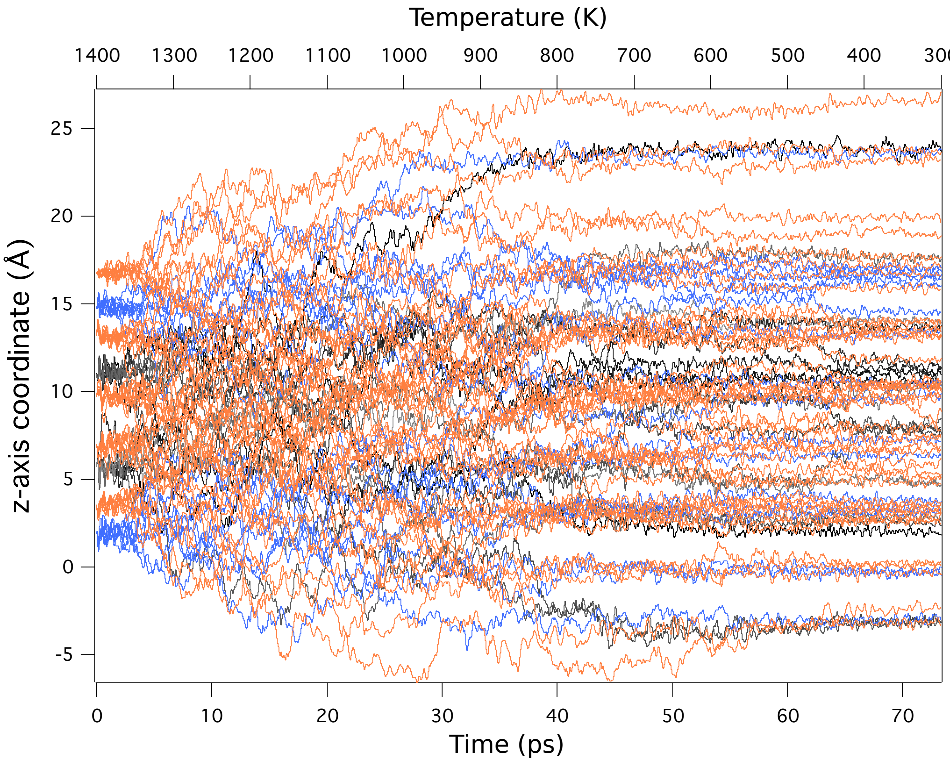

Supplementary Figure 5. The z-coordinate of atoms in the unstrained superlattice structure when cooled from 1400 K to 300 K. The layered structure is lost within 5 ps. The layers interdiffuse and upon crystallization form disorganised layers containing a mixture of Ge-Te-Sb atoms. The layers are unstable at this high temperature. The atoms intermix resulting in elementally mixed atomic layers. Colour code: Te –Orange, Sb –blue, Ge –black.

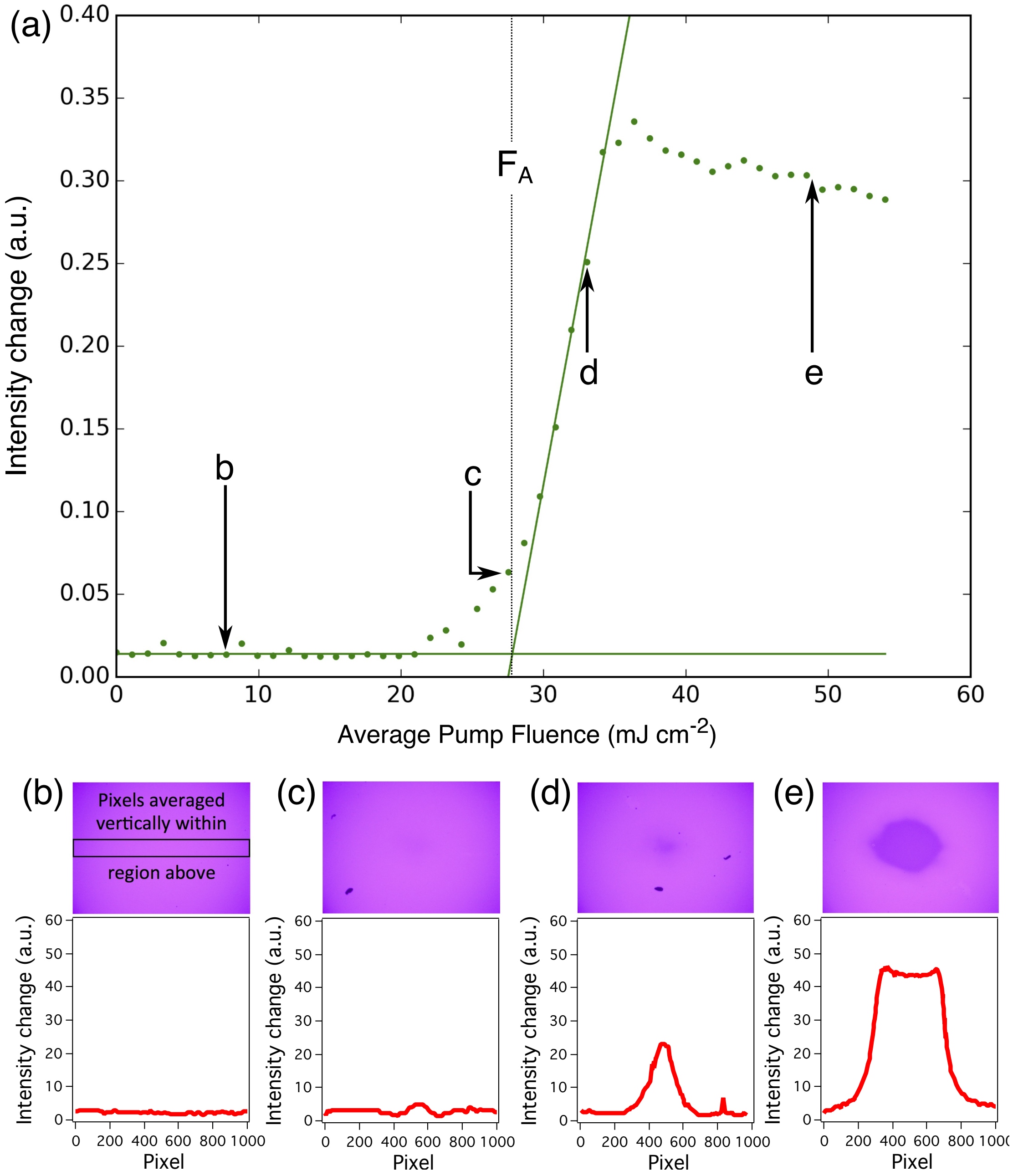

Supplementary Figure 6. A typical reflectivity measurement for the femtosecond laser switched spots in the Sb_2Te_3–GeTe superlattice samples. (a) shows the average reflectivity of the irradiated area as a function of the average laser pump fluence. A linear fit across the fluence range where the sample's reflectivity increases is extrapolated to determine the minimum fluence, F_A, necessary to switch. The lower panels show microscope images of the laser irradiated areas and their associated change in reflected intensity line profiles. Areas laser switched with fluences (b) 8 mJ cm^{-2}, (c) 28 mJ cm^{-2}, (d) 33 mJ cm^{-2}, and (e) 49 mJ cm^{-2} are shown. For reference these fluences are marked in panel (a) with letters b-e respectively.

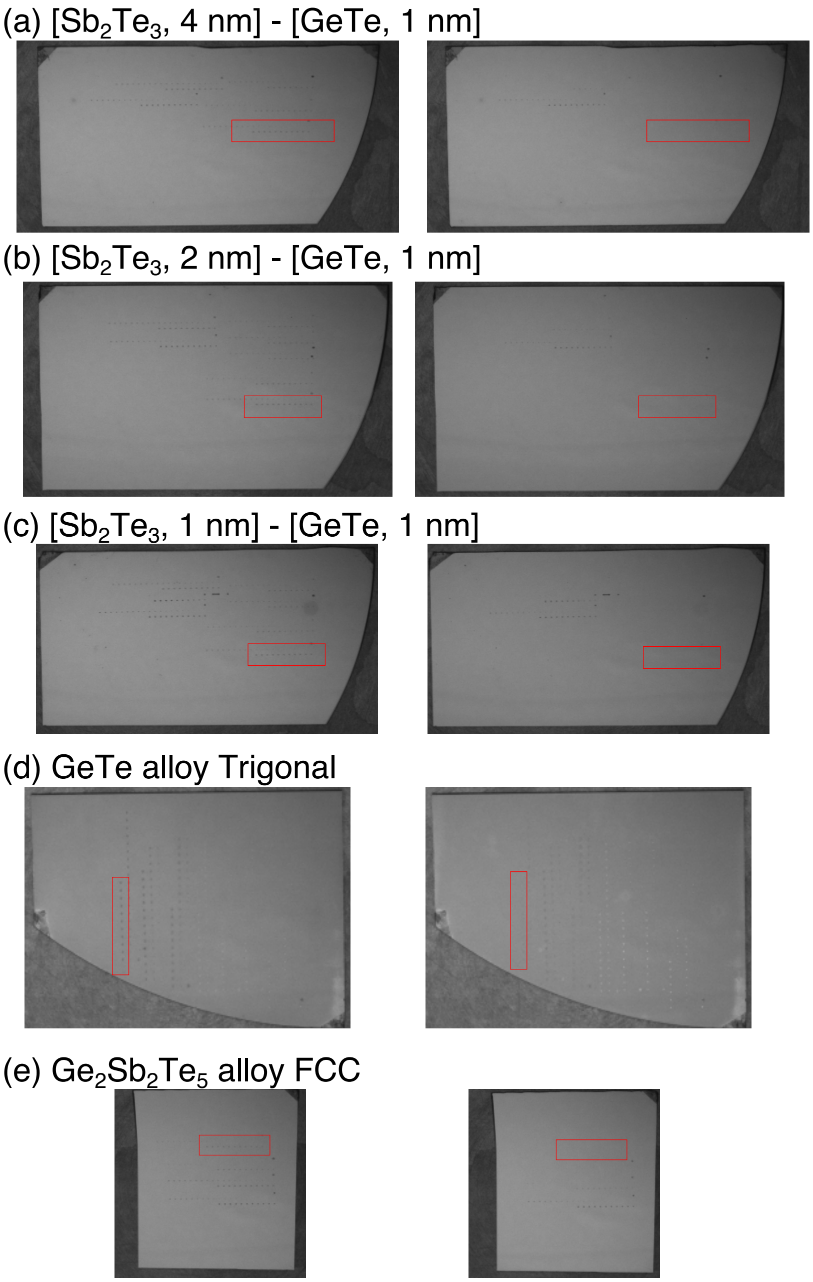

Supplementary Figure 7. Recrystallisation of Sb_2Te_3–GeTe superlattice samples. The lefthand images show the laser switched marks, whilst the righthand images show the same area after annealing at 220 °C. Images a–c show superlattice samples, whilst (d) shows GeTe and (e) shows $Ge_2Sb_2Te_5$ samples, which have been included for reference. These samples were used to measure the minimum fluence necessary to switch the material into a low reflectivity state. The threshold fluence, F_A for this type of switching behaviour is given in Fig. 3a of the main manuscript.

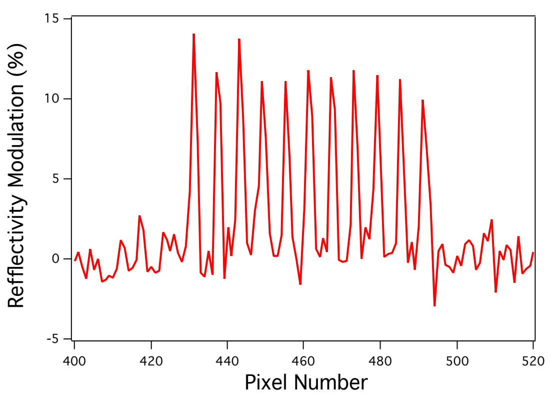

(a) Sb_2Te_3, 4 nm – GeTe, 1 nm

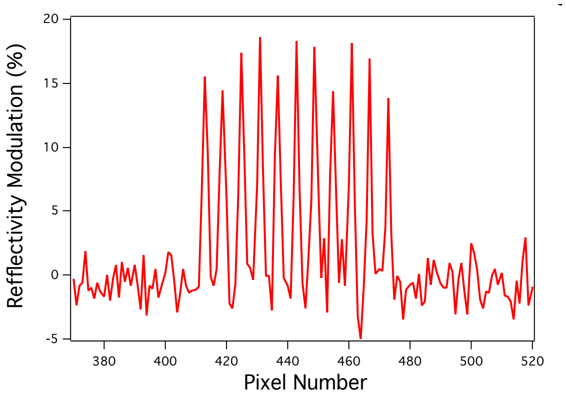

(b) Sb_2Te_3, 2 nm – GeTe, 1 nm

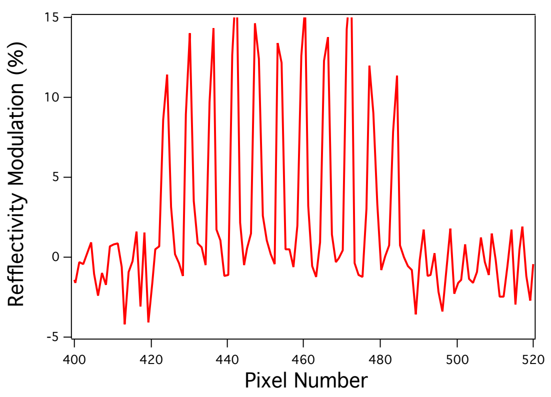

(c) Sb_2Te_3, 1 nm – GeTe, 1 nm

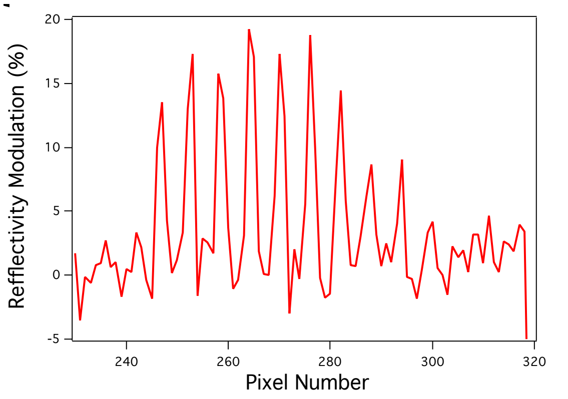

(d) Trigonal GeTe

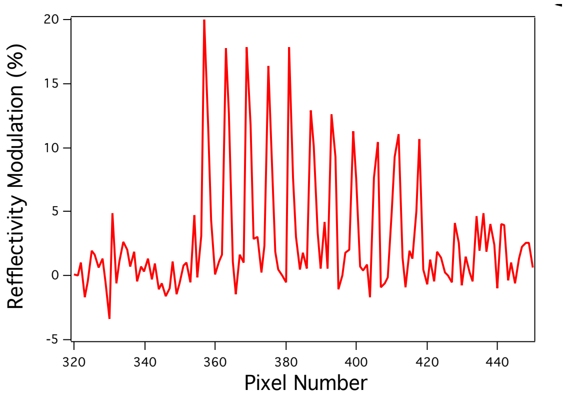

(e) Cubic $Ge_2Sb_2Te_5$

Supplementary Figure 8. Recrystallisation reflectivity modulation line profiles. The reflectivity modulation of the fs laser switched marks after annealing at 220 °C are presented for the superlattice samples (a-c), a [00L] oriented GeTe film (d), and cubic $Ge_2Sb_2Te_5$ (e). The line profiles correspond to the area indicated by the red box in Supplementary Figure 7.

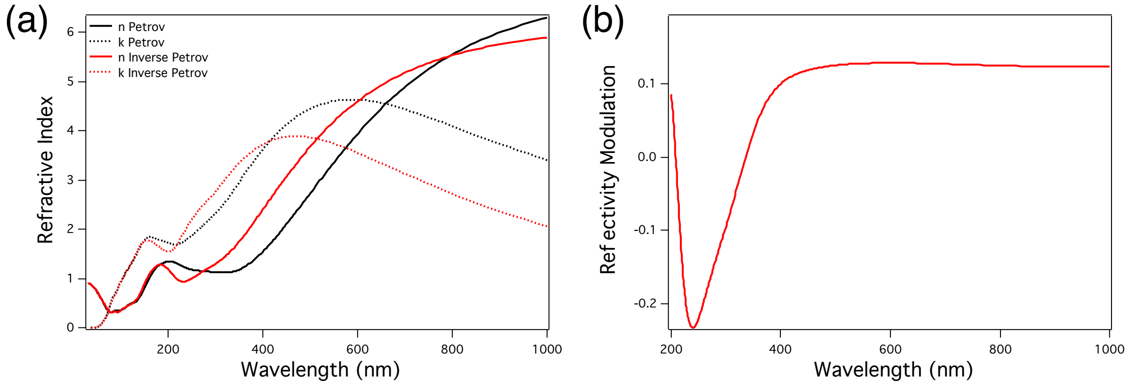

Supplementary Figure 9. Modelled refractive index and corresponding reflectivity modulation for the Sb_2Te_3–GeTe superlattice. (a) the real, n, and imaginary, k, components of the refractive index for the Ge atoms in the Petrov and inverse Petrov configurations. (b) the reflectivity modulation across the visible and near infrared spectral range. See Supplementary Note 6 for the calculation details

SUPPLEMENTARY TABLES

Temp (K)	Strain (%)					
	0.0	0.2	0.5	0.9	1.5	2.0
700	-	-	-	-	-	-
800	-	-	-	-	-	1
900	-	-	-	-	2	7
1000	-	-	-	-	8	9
1100	-	-	-	6	melts	10
1200	-	-	-	melts	melts	melts
1300	-	-	melts	melts	melts	melts
1400	melts	melts	melts	melts	melts	melts

Supplementary Table 1: The number of atoms switched at the end of 5 ps density functional molecular dynamics simulations. Each layer has 9 atoms, and there are two Ge-layers in the structure. If the structure is disordered enough to make layers indistinguishable then it is categorised as melting.

SUPPLEMENTARY NOTES

Supplementary Note 1: Sb_2Te_3–GeTe superlattice structure measurements

Bragg-Brentano symmetric x-ray diffraction scans were taken from the superlattice samples, with a Bruker D8 advance system. Only (00L) peaks are present in the XRD patterns from the superlattices (see Supplementary Figure 1), which indicates that the materials are grown with the desired layer structure.

The hexagonal lattice constants for bulk GeTe given in Table I of the main text were obtained from ref.1. As previously reported[2,3], the GeTe layer is grown along the Sb_2Te_3 – GeTe superlattice [111] direction of its rhombohedral primitive cell, which is aligned to the c-axis of a hexagonal Sb_2Te_3 crystal. Supplementary Figure 2 is helpful to visualise the equivalence between the lattice vectors for rhombohedral and hexagonal settings.

The in-plane lattice parameter of the hexagonal cell is equivalent to the $a = b = c$ lattice vector of the Sb_2Te_3 rhombohedral primitive cell. In the superlattice structure, the Sb_2Te_3 layers act as a template for growth along the [111] direction of the GeTe rhombohedral cell. We therefore describe the GeTe unit cell using its hexagonal setting, where $a = 4.16$ Å and $c = 10.66$ Å for bulk GeTe.

The superlattices were grown with a fibre-like texture with strong (00L) preferred orientation. The crystallographic domain size was typically 150 nm, which was measured by scanning electron microscopy and atomic force microscopy. Since the superlattice layer thicknesses are much smaller than the crystal domain size, the stresses in the superlattice layers are dominated by the mismatch between the layer thicknesses rather than domain edge effects. Thus it seems that long range in-plane crystallographic order is not absolutely necessary for growing in-domain strained layers, and industrially scalable growth techniques, such as sputtering, are useful for growing strained vdW superlattice heterostructures.

Supplementary Note 2: Premelt disordering

Supplementary Figure 3 shows the crystallinity, of the Sb_2Te_3 and GeTe layers within the vdW superlattice heterostructure as a function of temperature whilst heating the material from 450 K to 1300 K at a rate of 1.97 $K\,ps^{-1}$. The GeTe layer's crystallinity is lowered to temperatures below that of the melting temperature of Sb_2Te_3. This shows that the GeTe layer is substantially less stable than the Sb_2Te_3 in the superlattice structure. In contrast the bulk melting temperature of GeTe is greater than that of Sb_2Te_3. From Table I in the main manuscript we see that the GeTe layer is strained by the Sb_2Te_3 in the superlattice structure. We also know that the resonant bonds in crystalline GeTe are sensitive to lattice distortions. Here the Sb_2Te_3 layer is distorting the GeTe crystal structure, which decreases the average overlap of p-orbital bonds in the GeTe and weakens resonant bonding. Thus the strained GeTe layer is more sensitive to distortions caused by phonons and its disordering temperature is lowered.

Supplementary Note 3: Diffusive atomic switching map

The response of Sb_2Te_3–GeTe structure to a heat pulse was modelled as a function of in-plane biaxial strain. Isothermal 5 ps runs were used to find the temperature threshold for switching at least one Ge atom into the vdW gap. Supplementary Table 1 shows the number of Ge atoms that switched into the vdW gap for DF/MD runs conducted at different temperatures and for different amounts of in-plane biaxial strain. The data in Supplementary Table 1 was used to map the strain–temperature region for GeTe disordering, which is shown in Fig. 3b in the main text. Since the atomic switching is stochastic in nature, we drew somewhat blurred boundaries between the different regions.

Supplementary Note 4: Superlattice atom trajectories

When no strain is applied to the superlattice we find that all atoms remain in their initial superlattice layers. See Supplementary Figure 4, which shows the z-axis position of the atoms as a function of time. The lines do not cross, which indicates that the material does not melt or exhibit Ge–Te diffusive atomic disordering. In contrast, at higher temperatures the material melts. Supplementary Figure 5 shows a similar type of plot for the same initial structure where the MD run was conducted at a temperature of 1400 K, which is above the material's melting temperature. The layer structure is quickly lost. After 10 ps there is substantial inter-diffusion of atomic species across the layers. As the material is cooled below 900 K the atoms crystallise but the layers are disordered and not elementally pure. We see from Fig. 3b in the main manuscript that at least 0.5 % strain is required for atomic switching of Te and Ge. Indeed, Fig. 3d, in the main manuscript, shows Ge–Te diffusive atomic switching when 1.5% strain is applied to the simulation at 950 K.

We find that if the structures melt and the layers inter-diffuse, then the melt tends to crystallise into layers but each layer can contain a mixture of Sb, Te and Ge atoms and the final structure resembles the trigonal phase of a typical $Ge_2Sb_2Te_5$ alloy. The separation of GeTe and Sb_2Te_3 layers is lost.

Supplementary Note 5: Switching of the suplerlattice samples

The switching energy of the Sb_2Te_3–GeTe superlattices was analysed by measuring the change in reflectivity after pumping the samples with a single 30 fs laser pulse. The threshold fluence to initiate a change in the samples' reflectivity was fit linearly to the fluence range where the reflectivity of the sample is increasing (i.e. after threshold and before saturation). Supplementary Figure 6a shows a typical data set and the fit, which is extrapolated to find the threshold fluence, F_A. Supplementary Figure 6 (b-e) show the microscope images, which were collected in reflectivity mode, for the corresponding sample. The dark patch in the centre of the image is the laser switched region. The charts below the images show the pixel intensity of the microscope camera. The intensity profile was averaged over a 30 pixel wide band and used to determine the reflectivity intensity change for each fluence.

To demonstrate the reversibility of the switched state we heated the laser switched samples on a hotplate to 220 °C at 2 °C/min. Photographs of the samples were taken before and after heating, see Supplementary Figure 7. Photos on the left shows the laser switched marks, which are visible as a dark (low reflectivity) spots. The righthand photos show the same area of the sample after heating. The red box, shows the laser switched marks that were analysed. After heating the low reflectivity laser switched marks disappear due to recrystallisation of the disordered GeTe layers. Marks that do not disappear are due to laser induced damage/ablation of the superlattice. We also analysed the reflection change of the laser irradiated spots after recrystallisation by measuring the change in the reflected light pixel intensity. Supplementary Figure 8 shows reflection change line profiles for the laser marks highlighted in the red box of Supplementary Figure 7. The reflectivity modulation percentage was calculated according to $R = 100\frac{I_c - I_d}{I_d}$, where I_c is the pixel intensity of the annealed bitmap, and I_d is pixel intensity of the laser switched bitmap. We see that as we scan over pixels that detect the laser switched area, the reflectivity increases by between 10% and 15% after recrystallisation. These results demonstrate that the Sb_2Te_3-GeTe superlattices can switch reversibly.

Supplementary Note 6: Calculated reflectivity modulation of superlattice

To confirm that the reflectivity modulation measured in Supplementary Figure 8 is reasonable, we used the CASTEP DFT code to compute the optical properties of the Sb_2Te_3–GeTe structure with the Ge atoms in the GeTe layer, which is commonly named the Petrov configuration, and the Ge atoms in the vdW gap, which is usually named the Inverted Petrov configuration[4]. The structures were optimised into the ground state using the GGA functional with the PBE exchange. The cut-off energy was 440 eV, whilst the SCF energy tolerance was 5×10^{-7} eV. The atomic positions and cell geometry were optimised to an energy tolerance better than 5×10^{-6} eV, and the maximum force was less than 0.1 eV/atom. We used a $7 \times 7 \times 2$ Monkhorst-Pack grid for the k-points. After fully relaxing the structures we calculated the band structure using the HSE03 exchange and a band energy tolerance better than 1×10^{-5} eV. Then the refractive index of the structures was computed by first calculating the absorption spectra assuming unpolarised light, and then using a Kramers-Kronig transformation to get the refractive index real part.

The structure shows a reflectivity modulation of approximately 10% across the visible spectrum. This change in reflectivity agrees well with the experimentally observed changes shown in Supplementary Figure 8.

SUPPLEMENTARY REFERENCES

[1] Shelimova, L., Karpinskii, O., Avilov, E. & Kretova, M. Crystal Structure, Phase Transitions, and Mechanical Properties of GeTe-Based Solid Solutions in the GeTe-PbTe-MTe Systems (M= Mn, Sc, La). *Inorg. Mat.* **29**, 1291–1298 (1993).

[2] Simpson, R. E. *et al.* Interfacial Phase-Change Memory. *Nature Nanotech.* **6**, 501 – 505 (2011).

[3] Simpson, R. E., Fons, P., Kolobov, A. V., Krbal, M. & Tominaga, J. Enhanced crystallization of GeTe from an Sb_2Te_3 template. *App. Phys. Lett.* **100**, 021911 (2012).

[4] Tominaga, J., Kolobov, A., Fons, P., Nakano, T. & Murakami, S. Ferroelectric Order Control of the Dirac-Semimetal Phase in GeTe-Sb_2Te_3 Superlattices. *Advanced Materials Interfaces* **1** (2014).
